# Association between pre-diagnostic dietary copper, zinc, and copper-to-zinc ratio and severity of ovarian cancer

**DOI:** 10.3389/fnut.2022.1003675

**Published:** 2022-11-15

**Authors:** Jia-Li Yin, Tao Tao, Zhao-Yan Wen, Ran Wang, Ming-Hui Sun, Chang Gao, Yu-Jiao Chang, Shi Yan, Xue Qin, Yu-Hong Zhao, Lan Wang, Song Gao

**Affiliations:** ^1^Department of Clinical Epidemiology, Shengjing Hospital of China Medical University, Shenyang, China; ^2^Clinical Research Center, Shengjing Hospital of China Medical University, Shenyang, China; ^3^Key Laboratory of Precision Medical Research on Major Chronic Disease, Shengjing Hospital of China Medical University, Shenyang, China; ^4^Department of Obstetrics and Gynecology, Shengjing Hospital of China Medical University, Shenyang, China

**Keywords:** ovarian cancer, severity, diet, copper, zinc, copper-to-zinc ratio

## Abstract

**Background:**

The impact of dietary trace elements intake on ovarian cancer (OC) severity is unknown.

**Objective:**

We firstly explore the relationship between dietary copper (Cu), zinc (Zn), and copper-to-zinc (Cu/Zn) ratio and severity of OC.

**Methods:**

This cross-sectional study included 701 women from the OC follow-up study between 2015 and 2020. Dietary information was collected by a validated food frequency questionnaire (FFQ). The severity information of OC including age at diagnosis, histological type, International Federation of Gynecology and Obstetrics (FIGO) stage, and histopathologic grade was ascertained from medical records. Logistic regression model was used to estimate the odds ratios (ORs) and 95% confidence intervals (CIs) of aforementioned associations.

**Results:**

Among 701 participants, the number of patients age at diagnosis older than 50 were 443 (63.2%). The number of patients diagnosed as serous, III–IV stage, and poorly differentiation OC were 477 (68.05%), 336 (47.93%), and 597 (85.16%), respectively. In addition, compared with the lowest tertile intake, higher possibility of non-serous OC was associated with the pre-diagnosis dietary Cu (OR = 2.39, 95% CI = 1.28–4.47, p trend < 0.05) and Cu/Zn ratio (OR = 2.06, 95% CI = 1.26–3.39, P trend < 0.05) in the highest tertile intake. The risk of poorly differentiation OC at diagnosis was significant inversely related to dietary Cu intake (OR = 0.40, 95% CI = 0.18–0.88, P trend < 0.05). Besides, the results of subgroup analyses were consistent with the main findings but not all of them showed statistical significance.

**Conclusion:**

Pre-diagnostic dietary Cu and Cu/Zn ratio were contributed to reducing the severity of OC at diagnosis, especially for the risk of serous OC and poorly differentiation OC.

## Graphical Abstract

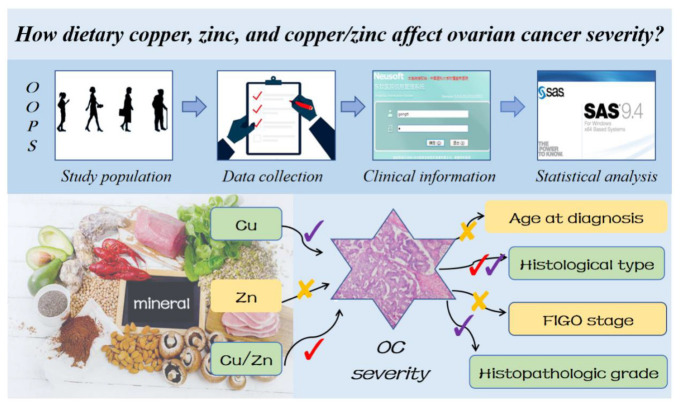

## Introduction

Ovarian Cancer (OC) ranks as a leading barrier to increasing the life expectancy of women worldwide (1). According to the latest statistics in 2020, about 314 thousand women were diagnosed with OC and 207 thousand women died of this disease in 185 countries (1). In China, the increasing incidence and mortality rate make OC the third most common gynecological malignancy (2). Owing to its unfavorable anatomy and a lack of effective screening strategies, OC is often diagnosed at an advanced stage (3). Specifically, most serous OC cases were diagnosed at stage III or IV with a survival rate lower than 43%, while the survival rate of non-serous OC was above 66% (4). Similarly, women who died of OC were older on average, and more likely to be diagnosed with serous, stage III-IV tumors (5). Therefore, age at diagnosis, histological type, International Federation of Gynecology and Obstetrics (FIGO) stage, and histopathologic grade are important factors reflecting the severity and prognosis of OC (6–10).

Dietary, as a key modifiable risk factor, can be used to formulate recommendations for the prevention of cancers, including OC (11–13). High-quality diets were inversely associated with the risk and development of OC (14, 15). Therefore, exploring relevant nutritional factors and looking for effective interventions are crucial to reducing the severity of OC. A growing body of evidence indicated that copper (Cu) and zinc (Zn) played vital roles in a series of anticancer processes that proceed through various mechanisms (16, 17). Cu serves as a limiting factor for multiple aspects of tumor progression, including growth, angiogenesis, and metastasis (18, 19). Zn is required for the catalytic activity of more than 200 enzymes, which are essential in immune function, cell divisions, and anti-tumor actions (20, 21). Besides, Caglayan et al. revealed that Cu and Zn-superoxide dismutase might protect cells against the biological damage of oxidative stress induced by reactive oxygen species (22). To date, it has been shown that serum Cu, Zn level, and copper-to-zinc (Cu/Zn) ratio can be used as diagnosis and prognostic indicators for some cancers (23–25). Of these, a case-control study in northeast China suggested that the serum Cu level and the Cu/Zn ratio were effective predictive indicators of lung cancer and might help to evaluate the prognosis of patients with non-small cell lung cancer (23). In the development of colorectal cancer, Stepien et al. (24) suggested that Cu level in relation to Zn (Cu/Zn ratio) became imbalanced in a cohort study. Furthermore, another cohort proved that higher serum Cu and Cu/Zn ratio might be associated with worse hepatocellular carcinoma survival (25). Kazi et al. (26) showed that Cu isotopes and plasma trace elements might serve as suitable biomarkers of thyroid cancer diagnosis. Of note, one meta-analysis and Mendelian randomization study showed that lower circulating Zn might be causally associated with higher OC risk, while no causal effect of circulating Cu on OC risk was found (27).

Currently, no previous study has explored the associations between dietary Cu, Zn, and Cu/Zn ratio and OC severity. Therefore, to firstly elucidate this topic, we carried out this study based on the data from the ovarian cancer follow-up study (OOPS) in 701 OC patients.

## Materials and methods

### Study design and population

**FIGURE 1:**
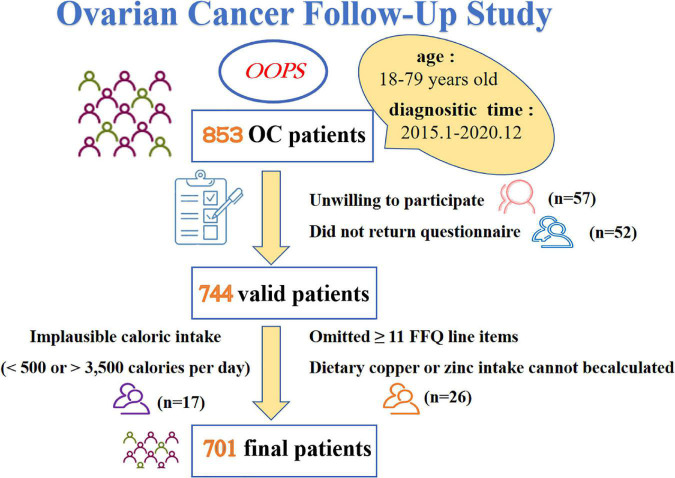
Flow chart of participants through study.

Patients included in this cross-sectional study were acquired from the OOPS (28), which is a prospective longitudinal cohort study of patients newly diagnosed with OC. Being approved by the Institutional Review Board of the Ethics Committee of Shengjing Hospital of China Medical University, Shenyang, China (2015PS38K), the OOPS was devoted to collecting clinical and lifestyle information and exploring their associations with cancer-related outcomes (29–34). Patients recruited during the baseline survey were histologically confirmed OC diagnosis, with age between 18 and 79 years old. We have diagnosed 853 patients with OC from January 2015 to December 2020, but only 796 women (93%) consented to participate and 744 (87%) women returned the completed study questionnaire. In addition, we excluded patients who were ineligible for analysis, such as implausible caloric intake was reported (< 500 or > 3,500 calories per day, *n* = 17) (35, 36), greater than or equal to 11 (10%) food frequency questionnaire (FFQ) line items were omitted (*n* = 24). Considering the necessity of Cu and Zn content for this study, we further excluded those whose dietary Cu or Zn intake cannot be calculated (*n* = 2). Finally, a total of 701 (82%) women contributed to the analyses (Figure 1).

### Data collection

Self-administered questionnaires were applied for collecting all related characteristics of one year before diagnosis, such as basic personal data, dietary features, and sleep status. Anthropometrics including weight and height were measured at baseline, which were used to calculate body mass index (BMI). Besides, clinical information concerning the age at diagnosis, histological type, FIGO stage, histopathologic grade, and comorbidities was collected from the electronic medical records of the Shengjing hospital information system. After the patient finished the baseline survey, clinical specialists extracted patient medical data from the information system at Shengjing Hospital every 6 months.

### Dietary exposure assessment

Our FFQ consisting of 111 items was used to assess dietary intake, which has been verified to have reasonable reliability and validity (29–31). The reproducibility coefficients (Spearman correlation coefficients and intraclass correlation coefficients) were above 0.5 for most food groups, and correlation coefficients (Spearman correlation coefficients) were between 0.3 and 0.7 for most food groups between the FFQ and weighed dietary records. Patients were required to recall their daily intake of these food items during the year before diagnosis. The dietary intake was assessed in times, including more than two times per day, one or two times per day, four to six times per week, two or three times per week, one time per week, two or three times per month, and almost never. To gather complete data on these items, face-to-face examinations on the self-administered questionnaires were carried out by well-trained researchers. Based on the Chinese Food Composition Tables (37), our nutrient intake was calculated by multiplying the frequency of consumption of each food by the nutrient content of the specified portions. Ultimately, we decided to select dietary Cu, Zn, and Cu/Zn ratio as our exposure to explore.

### Outcome selection

Age at diagnosis, histological type, FIGO stage, and histopathologic grade are important factors reflecting the severity and prognosis of OC (6–10). Our primary outcome was the severity of OC, defined as a composite of some typical clinical characteristics containing age at diagnosis (< 50 or ≥ 50 years), histological type (serous or non-serous), FIGO stage (I – II or III – IV) serous or ical clintopathologic grade (well, moderately, and poorly differentiated) as our study outcome.

### Statistical analysis

Baseline characteristics were described according to the tertiles of dietary Cu, Zn, and Cu/Zn ratio intake, where the lowest tertile served as the reference group. Descriptive statistics were performed to observe and distinguish individual features among groups. Continuous variables were described by the mean ± standard deviation, and categorical variables were exhibited using counts and percentages. Differences in general characteristics among groups were assessed with the one-way ANOVA or the Kruskal-Wallis test, except for categorical variables, which were assessed with the χ^2^-test. Logistic regression analyses were used to calculate the odds ratios (ORs) and 95% confidence intervals (CIs) for the association of dietary Cu, Zn, and Cu/Zn ratio intake with the severity of OC. The linear trend of cross-increasing tertiles was tested using the median value of each tertile as a continuous variable based on logistic regression.

The selection of covariates for the multivariable model was based on the degree of correlation with the exposure, clinical significance, and similar articles (25, 38, 39). Model 1 was adjusted for age at diagnosis (< 50 and ≥ 50 years), BMI (continuous, kg/m^2^), education (junior secondary or below, senior high school or technical secondary school, and junior college or university or above), income (< 5,000, 5,000– < 10,000, and ≥ 10,000), smoking status (yes or no), drinking status (yes or no), menarche age (≤ 16 and > 16), menopausal status (yes or no), physical activity (continuous, metabolic equivalent task/hours/days), and parity (≤ 1 and ≥ 2). We also applied a model adjustment for total energy intake (continuous, kcal), comorbidities (yes or no), diet change (yes or no), dietary protein intake (continuous, g/day), and dietary fiber intake (continuous, g/day) based on model 1. In the third model, dietary calcium intake (continuous, mg/day), and dietary iron intake (continuous, mg/day) were further adjusted based on model 2. Multivariate logistic regression collinearity diagnostic analysis was performed for adjustment models and no collinearity between variables was found.

In addition, we calculated adjusted OR and 95%CI in subgroup analyses and interaction analyses stratified by menopausal status (“no” compared to “yes”), BMI (< 25 compared to ≥ 25 kg/m^2^), and comorbidities status (“no” compared to “yes”). Finally, a restricted cubic spline model was applied to examine whether there was a non-linear relationship between exposures and outcomes. All statistical analyses were performed using SAS Version 9.4 (SAS Institute, Cary, NC, USA). All *P*-values were two-tailed and the difference was defined to be significant when *P* < 0.05.

## Results

Table 1 presented the basic characteristics of the 701 OC patients by tertiles of dietary Cu and Zn intake. Among all patients, about 71.90% gave birth less than or equal to one time, while only 28.10% gave birth more than once. Besides, there were 59.92% patients had an average monthly income of lower than 5,000 yuan, 27.53% patients had an average monthly income of 5,000–10,000 yuan, and only 12.55% patients had an average monthly income of over than 10,000 yuan. The distribution of parity and income significantly differed by tertiles of dietary Cu and Zn, respectively (all *P* < 0.05). Moreover, socio-demographic and lifestyle status of 701 patients were described in Table 2, on the basic of tertiles of dietary Cu/Zn ratio. In summary, OC patients who had a higher intake of Cu, Zn, or Cu/Zn ratio tended to have higher total energy, dietary protein, fiber, calcium, and iron intake (all *P* < 0.05).

**TABLE 1:** Baseline characteristics of ovarian cancer patients by tertiles of dietary copper and zinc intake (*N* = 701).

Characteristics	All patients	Tertiles of dietary copper intake			*P*-value*	Tertiles of dietary zinc intake			*P*-value*
		I	II	III		I	II	III	
Range (mg/d)		<1.30	1.30–2.37	>2.37		<6.98	6.98–9.61	>9.61	
No. of patients/deaths	701/130	233/47	233/45	235/38	0.50	233/43	233/45	235/42	0.92
Mean (SD) age at diagnosis (years)	53.60 (9.44)	54.19 (9.25)	52.75 (9.72)	53.85 (9.32)	0.52	53.86 (9.61)	53.37 (9.26)	53.56 (9.48)	0.78
Mean (SD) body mass index (kg/m^2^)	23.24 (3.59)	23.11 (3.30)	23.12 (3.52)	23.51 (3.92)	0.60	23.06 (3.61)	23.12 (3.83)	23.55 (3.30)	0.12
Mean (SD) physical activity (MET h/d)	15.77 (11.25)	15.56 (11.35)	15.46 (10.81)	16.29 (11.61)	0.66	15.52 (11.28)	16.22 (11.75)	15.58 (10.75)	0.79
Ever cigarette smoking	68 (9.70)	22 (9.44)	25 (10.73)	21 (8.94)	0.80	24 (10.30)	20 (8.58)	24 (10.21)	0.78
Ever alcohol drinking	149 (21.26)	44 (18.88)	56 (24.03)	49 (20.85)	0.39	45 (19.31)	46 (19.74)	58 (24.68)	0.29
Ever menopause	506 (72.18)	170 (72.96)	166 (71.24)	170 (72.34)	0.92	178 (76.39)	160 (68.67)	168 (71.49)	0.17
Ever diet change before diagnosis	168 (23.97)	45 (19.31)	55 (23.61)	68 (28.94)	0.05	48 (20.60)	53 (22.75)	67 (28.51)	0.12
Parity					<0.05				0.19
≤ 1	504 (71.90)	154 (66.09)	170 (72.96)	180 (76.60)		158 (67.81)	169 (72.53)	177 (75.32)	
≥ 2	197 (28.10)	79 (33.91)	63 (27.04)	55 (23.40)		75 (32.19)	64 (27.47)	58 (24.68)	
Menarche age					0.44				0.58
≤ 16	591 (84.31)	191 (81.97)	201 (86.27)	199 (84.68)		192 (82.40)	200 (85.84)	199 (84.68)	
> 16	110 (15.69)	42 (18.03)	32 (13.73)	36 (15.32)		41 (17.60)	33 (14.16)	36 (15.32)	
Educational level					0.11				0.20
Junior secondary or below	373 (53.21)	135 (57.94)	117 (50.22)	121 (51.49)		131 (56.22)	127 (54.50)	115 (48.94)	
Senior high school/technical secondary school	147 (20.97)	43 (18.45)	60 (25.75)	44 (18.72)		46 (19.74)	54 (23.18)	47 (20.00)	
Junior college/university or above	181 (25.82)	55 (23.61)	56 (24.03)	70 (29.79)		56 (24.04)	52 (22.32)	73 (31.06)	
Income per month (Yuan)					0.58				<0.05
< 5,000	420 (59.92)	147 (63.09)	142 (60.94)	131 (55.74)		152 (65.24)	142 (60.94)	126 (53.62)	
5,000– < 10,000	193 (27.53)	58 (24.89)	63 (27.04)	72 (30.64)		48 (20.60)	66 (28.33)	79 (33.62)	
≥ 10,000	88 (12.55)	28 (12.02)	28 (12.02)	32 (13.62)		33 (14.16)	25 (10.73)	30 (12.76)	
Mean (SD) total energy intake (kcal/d)	1456.93 (552.98)	1013.10 (276.58)	1452.73 (354.13)	1901.13 (565.89)	<0.05	959.80 (228.92)	1403.83 (276.55)	2002.46 (489.07)	<0.05
Mean (SD) protein intake (g/d)	58.51 (24.79)	37.73 (10.54)	57.09 (14.56)	80.53 (24.48)	<0.05	36.35 (9.09)	54.49 (10.90)	84.47 (21.38)	<0.05
Mean (SD) total fiber intake (g/d)	17.53 (8.62)	10.53 (3.79)	16.53 (4.67)	25.46 (8.55)	<0.05	11.25 (4.37)	16.10 (5.19)	25.17 (8.75)	<0.05
Mean (SD) calcium intake (mg/d)	454.47 (248.46)	260.25 (107.62)	428.87 (135.14)	672.41 (262.99)	<0.05	265.16 (105.25)	417.40 (146.59)	678.91 (254.43)	<0.05
Mean (SD) iron intake (mg/d)	18.92 (7.98)	12.21 (2.84)	18.26 (4.30)	26.22 (8.08)	<0.05	11.92 (2.59)	17.62 (3.18)	27.14 (7.44)	<0.05

Values are numbers (percentages) unless stated otherwise. MET, metabolic equivalents of task; SD, standard deviation. *P-values were determined with one-way ANOVA or the Kruskal-Wallis test for continuous variables and the chi-square test for categorical variables.

**TABLE 2:** Baseline characteristics of ovarian cancer patients by tertiles of dietary copper/zinc ratio (*N* = 701).

Characteristics	All patients	Tertiles of dietary copper/zinc ratio			*P*-value*
		I	II	III	
Range (mg/d)		<0.18	0.18–0.26	>0.26	
No. of patients/deaths	701/130	233/46	233/48	235/36	0.29
Mean (SD) age at diagnosis (years)	53.60 (9.44)	54.34 (9.20)	52.80 (9.34)	53.65 (9.74)	0.25
Mean (SD) body mass index (kg/m^2^)	32.79 (16.54)	23.17 (3.33)	23.16 (3.29)	23.40 (4.09)	0.94
Mean (SD) physical activity (MET h/d)	15.78 (11.25)	15.77 (11.41)	15.80 (10.89)	15.74 (11.50)	0.95
Ever cigarette smoking	68 (9.70)	25 (10.73)	21 (9.01)	22 (9.36)	0.80
Ever alcohol drinking	149 (21.26)	50 (21.46)	50 (21.46)	49 (20.85)	0.98
Ever menopause	506 (72.18)	172 (73.82)	165 (70.82)	169 (71.91)	0.76
Ever diet change before diagnosis	168 (23.97)	49 (21.03)	55 (23.61)	64 (27.23)	0.29
Parity					0.32
≤1	504 (71.90)	160 (68.67)	168 (72.10)	176 (74.89)	
≥2	197 (28.10)	73 (31.33)	65 (27.90)	59 (25.11)	
Menarche age					0.61
≤16	591 (84.31)	198 (84.98)	192 (82.40)	201 (85.53)	
>16	110 (15.69)	35 (15.02)	41 (17.60)	34 (14.47)	
Educational level					0.44
Junior secondary or below	373 (53.21)	133 (57.08)	119 (51.07)	121 (51.49)	
Senior high school/technical secondary school	147 (20.97)	49 (21.03)	52 (22.32)	46 (19.57)	
Junior college/university or above	181 (25.82)	51 (21.89)	62 (26.61)	68 (28.94)	
Income per month (Yuan)					0.11
<5,000	420 (59.91)	141 (60.52)	141 (60.52)	138 (58.72)	
5,000– < 10,000	193 (27.53)	71 (30.47)	54 (23.18)	68 (28.94)	
≥ 10,000	88 (12.56)	21 (9.01)	38 (16.30)	29 (12.34)	
Mean (SD) total energy intake (kcal/d)	1456.93 (552.98)	1253.53 (464.72)	1424.92 (471.72)	1690.33 (618.93)	<0.05
Mean (SD) protein intake (g/d)	58.51 (24.79)	47.21 (20.26)	56.40 (19.61)	71.82 (27.22)	<0.05
Mean (SD) total fiber intake (g/d)	17.53 (8.62)	12.27 (5.32)	17.04 (6.99)	23.23 (9.22)	<0.05
Mean (SD) calcium intake (mg/d)	454.47 (248.46)	333.55 (194.16)	427.52 (182.69)	601.07 (278.09)	<0.05
Mean (SD) iron intake (mg/d)	18.92 (7.98)	15.00 (5.61)	18.28 (6.71)	23.43 (8.86)	<0.05

Values are numbers (percentages) unless stated otherwise. MET, metabolic equivalents of task; SD, standard deviation. *P-values were determined with one-way ANOVA or the Kruskal-Wallis test for continuous variables and the chi-square test for categorical variable.

After controlling for potential confounders, we displayed the associations between dietary Cu, Zn, and Cu/Zn ratio and selected clinical outcomes in Table 3. Higher intake of dietary Cu was associated with a higher possibility to be diagnosed as non-serous OC, a linear trend was also evident (OR = 2.39, 95%CI: 1.28–4.47, P trend < 0.05). Besides, each 1-SD increment in dietary Cu was significantly associated with an increased non-serous OC incidence (OR = 1.33, 95%CI: 1.00–1.76). In addition, we observed that the association between dietary Cu/Zn ratio intake and non-serous OC were positive among different groups by tertiles (OR = 2.06, 95%CI: 1.26–3.39, P trend < 0.05). However, compared with the lowest tertile of dietary Cu, the risk of poorly differentiated OC was reduced in the patients with the highest tertile intake (OR = 0.40, 95%CI: 0.18–0.88, P trend < 0.05). Though we observe dietary Zn produced a borderline effect on histopathologic grade (OR = 0.39, 95%CI: 0.15–1.01, P trend = 0.05), each 1-SD increment in dietary Zn was significant inversely associated with the risk of poorly differentiated OC (OR = 0.44, 95%CI: 0.21–0.91). Furthermore, we did not find significant associations between dietary Cu, Zn, and Cu/Zn ratio levels and age at diagnosis or FIGO stage.

**TABLE 3:** Odds ratios (ORs) and 95% confidence intervals (CIs) for the association between dietary copper, zinc, and copper/zinc ratio intake and selected clinical characteristics among 701 ovarian cancer patients.

Characteristics	Dietary intake				
	Tertile I*	Tertile II*	Tertile III*	*P* trend^†^	Continuous^‡^
**Dietary copper**					
**Age at diagnosis (years), > 50 vs. ≤ 50**					
Model 1	1.00 (Ref)	0.77 (0.49–1.20)	1.01 (0.64–1.57)	0.68	0.99 (0.83–1.19)
Model 2	1.00 (Ref)	0.88 (0.53–1.45)	1.35 (0.69–2.69)	0.22	1.09 (0.81–1.48)
Model 3	1.00 (Ref)	0.85 (0.52–1.41)	1.25 (0.63–2.51)	0.32	1.05 (0.77–1.44)
**Histological type, non-serous vs. serous**					
Model 1	1.00 (Ref)	0.91 (0.60–1.37)	1.15 (0.77–1.71)	0.38	1.01 (0.85–1.19)
Model 2	1.00 (Ref)	1.24 (0.78–1.96)	2.17 (1.18–4.02)	<0.05	1.28 (0.97–1.67)
Model 3	1.00 (Ref)	1.26 (0.79–2.01)	2.39 (1.28–4.47)	<0.05	1.33 (1.00–1.76)
**FIGO stage, III–IV vs. I–II (Ref)**					
Model 1	1.00 (Ref)	1.09 (0.74–1.59)	1.11 (0.76–1.63)	0.61	1.04 (0.89–1.22)
Model 2	1.00 (Ref)	0.94 (0.62–1.44)	0.82 (0.46–1.45)	0.47	0.92 (0.71–1.19)
Model 3	1.00 (Ref)	0.94 (0.61–1.44)	0.80 (0.45–1.45)	0.45	0.95 (0.73–1.24)
**Histopathologic grade, poorly vs. moderately vs. well**					
Model 1	1.00 (Ref)	1.21 (0.69–2.12)	0.70 (0.42–1.18)	0.08	0.88 (0.72–1.08)
Model 2	1.00 (Ref)	0.92 (0.49–1.70)	0.41 (0.19–0.89)	<0.05	0.76 (0.55–1.04)
Model 3	1.00 (Ref)	0.92 (0.49–1.72)	0.40 (0.18–0.88)	<0.05	0.78 (0.56–1.08)
**Dietary zinc**					
**Age at diagnosis (years), > 50 vs. ≤ 50**					
Model 1	1.00 (Ref)	0.93 (0.60–1.44)	1.15 (0.73–1.80)	0.53	1.03 (0.86–1.23)
Model 2	1.00 (Ref)	1.08 (0.65–1.79)	1.66 (0.77–3.61)	0.22	1.26 (0.79–2.01)
Model 3	1.00 (Ref)	1.10 (0.66–1.84)	1.72 (0.79–3.83)	0.20	1.46 (0.81–2.65)
**Histological type, non-serous vs. serous**					
Model 1	1.00 (Ref)	0.81 (0.55–1.22)	0.77 (0.51–1.16)	0.21	0.89 (0.75–1.05)
Model 2	1.00 (Ref)	0.96 (0.60–1.52)	1.09 (0.54–2.19)	0.84	1.12 (0.73–1.74)
Model 3	1.00 (Ref)	0.90 (0.56–1.44)	0.92 (0.45–1.90)	0.80	0.81 (0.46–1.42)
**FIGO stage, III–IV vs. I–II**					
Model 1	1.00 (Ref)	1.25 (0.86–1.83)	1.20 (0.82–1.76)	0.36	1.07 (0.92–1.25)
Model 2	1.00 (Ref)	1.20 (0.78–1.85)	1.01 (0.53–1.93)	0.91	1.01 (0.68–1.52)
Model 3	1.00 (Ref)	1.27 (0.82–1.97)	1.15 (0.59–2.24)	0.63	1.46 (0.87–2.46)
**Histopathologic grade, poorly vs. moderately vs. well**					
Model 1	1.00 (Ref)	0.84 (0.49–1.44)	0.77 (0.45–1.31)	0.34	0.93 (0.76–1.15)
Model 2	1.00 (Ref)	0.58 (0.31–1.08)	0.34 (0.13–0.85)	<0.05	0.42 (0.23–0.76)
Model 3	1.00 (Ref)	0.61 (0.33–1.15)	0.39 (0.15–1.01)	0.05	0.44 (0.21–0.91)
**Dietary copper/zinc intake**					
**Age at diagnosis (years), > 50 vs. ≤ 50**					
Model 1	1.00 (Ref)	0.85 (0.54–1.32)	0.83 (0.53–1.29)	0.47	0.99 (0.83–1.19)
Model 2	1.00 (Ref)	0.91 (0.57–1.46)	0.91 (0.54–1.55)	0.78	1.06 (0.86–1.33)
Model 3	1.00 (Ref)	0.90 (0.57–1.45)	0.89 (0.52–1.52)	0.71	1.05 (0.85–1.32)
**Histological type, non-serous vs. serous**					
Model 1	1.00 (Ref)	1.04 (0.69–1.57)	1.46 (0.98–2.19)	<0.05	1.15 (0.98–1.36)
Model 2	1.00 (Ref)	1.14 (0.74–1.76)	1.86 (1.14–3.03)	<0.05	1.23 (1.01–1.52)
Model 3	1.00 (Ref)	1.19 (0.77–1.84)	2.06 (1.26–3.39)	<0.05	1.29 (1.06–1.59)
**FIGO stage, III–IV vs. I–II**					
Model 1	1.00 (Ref)	1.27 (0.87–1.86)	1.07 (0.74–1.56)	0.91	1.01 (0.86–1.18)
Model 2	1.00 (Ref)	1.17 (0.78–1.74)	0.87 (0.55–1.38)	0.40	0.94 (0.77–1.13)
Model 3	1.00 (Ref)	1.15 (0.77–1.72)	0.85 (0.53–1.35)	0.36	0.93 (0.76–1.13)
**Histopathologic grade, poorly vs. moderately vs. well**					
Model 1	1.00 (Ref)	1.13 (0.65–1.97)	0.71 (0.42–1.18)	0.11	0.84 (0.70–1.02)
Model 2	1.00 (Ref)	1.12 (0.63–2.00)	0.68 (0.37–1.27)	0.14	0.86 (0.69–1.06)
Model 3	1.00 (Ref)	1.05 (0.58–1.88)	0.60 (0.32–1.12)	0.06	0.83 (0.66–1.03)

CI, confidence interval; OR, Odd ratio; Ref, reference; FIGO, The International Federation of Gynecology and Obstetrics. *OR and 95% CI were calculated with the logistic regression model. ^†^Test for trend was based on variables containing the median value for each tertile. ^‡^Continuous intakes were calculated by dividing the original intake by the standard deviation (For 1.61 mg/d increments of dietary copper, 3.10 mg/d increments of dietary zinc, 0.13 increments of dietary copper/zinc ratio). Model 1 was adjusted for age at diagnosis, body mass index, education, income, menarche age, menopausal status, smoking status, drinking status, physical activity, and parity. Model 2 was further adjusted for diet change, and total energy, protein, and fiber intake based on model 1. Model 3 was further adjusted for dietary calcium and iron intake based on model 2.

The relationships between dietary Cu, Zn, and Cu/Zn ratio intake and the severity of OC were evaluated across potential effect modifying variables. No significant interactions were found in the subgroup analyses stratified by menopausal status, BMI, and comorbidity (Supplementary Tables 1–4). The direction of these results was mainly consistent with the main findings but not all of them showed statistical significance. Of note, in menopausal or no comorbidity patients, the lowest serous OC risk was associated with the highest dietary Cu or Cu/Zn ratio intake (Supplementary Table 1). Additionally, OC patients with a higher intake of dietary Cu or Zn were more likely to be diagnosed as well-differentiated OC in the subgroup of BMI < 25 kg/m^2^ and comorbidities (Supplementary Table 2). Besides, we did not find the non-linear relationships between dietary Cu, Zn, and Cu/Zn ratio intake and the severity of OC.

## Discussion

The present study with 701 OC patients reports, to our knowledge, the first one to study pre-diagnostic dietary Cu, Zn, and Cu/Zn and severity of OC to date. Our findings showed that higher dietary Cu and Cu/Zn ratio intake were related to patients diagnosed as non-serous OC, which means more diverse treatment options and longer survival time. This situation is more obvious in menopausal or no comorbidity patients. Moreover, in the women of BMI < 25 kg/m^2^ and comorbidities, high dietary Cu and Zn intake were accompanied by the emergence of well-differentiated OC with a lower severity. These findings were consistent with some studies (24, 26, 38, 40, 41) but different from others (23, 39, 42, 43).

### Comparison with other studies

Up to now, no prior study aimed to explore the association between pre-diagnosis intake of dietary Cu, Zn, and Cu/Zn ratio and the severity of OC. Additionally, epidemiological evidence of other tumors has been limited and inconsistent (23, 24, 26, 38–43). On the one hand, our findings on the Cu and Cu/Zn ratio were consistent with the results from Atakul et al. (38) but contrary to others (23, 39, 42, 43). For example, a cross-sectional study suggested that endometrial cancer patients exhibited lower serum Cu, Zn, and Cu/Zn ratio when compared with controls (38). However, a case-control study with 146 patients and 146 controls suggested that increased serum Cu level and Cu/Zn ratio might improve the prognosis of patients with non-small cell lung cancer (23). The findings from a cohort study in the US proved that serum Cu level and Cu/Zn ratio had negative effects on all-cause death risk in lung cancer patients (39). Furthermore, a case-control study indicated that higher serum Cu levels and elevated Cu/Zn ratio might serve as biomarkers for the increased severity of viral hepatic damage, which may progress into more serious pathological outcomes and hepatocellular carcinoma eventually (42). Besides, Li et al. (43) revealed that each 1-SD increase in ln-transformed plasma copper were significantly associated with increased cancer incidence. The reasons for inconsistency may be the diversity in sample size, study population, especially for study outcomes. On the other hand, our findings of Zn from subgroup analysis are in line with previous studies (24, 26, 40, 41). For example, two multicenter prospective cohort studies from Europe presented that a higher circulating concentration of Zn was inversely related to the risk of hepatocellular carcinoma and colorectal cancer (24, 40). Kazi et al. (26) showed that plasma concentrations of Zn significantly lower in thyroid cancer patients (*n* = 46) as compared to healthy controls (*n* = 50). A cross-sectional study in Nigeria revealed that low plasma Zn status was associated with severe grade and advanced prostate cancer (41).

### Potential biological mechanisms

Oxidative stress and trace elements have been established to be associated with cancer development (19, 21, 44). The role of Cu or Zn superoxide dismutase, whose functions are dependent on the adequate presence of both Cu and Zn, is to catalyze reactive oxygen species degradation originating from metabolic pathways to less reactive compounds, which are then removed by other antioxidant enzymes such as reduced glutathione peroxidase, thus reducing oxidative stress (22). In addition, cuproplasia is proved to be linked to a diverse array of cellular processes, including antioxidant defense, redox signaling, and autophagy (45, 46). Besides, Cu and Zn may control cancer development by maintaining normal cell cycle progression, inhibiting proliferation, tumor invasion, and inflammation (47). For example, an increased Cu requirement by prostate cancer cells because the presence of a mutant Cu-transporting Atp7b protein may change Cu-integration in serum and cause a remarkable reduction in prostate cancer burden and disease severity, abrogating adenocarcinoma development (48). Several studies showed that dysregulation of Zn transporters was implicated in the progression of cancer (49, 50). Furthermore, some researchers indicated Zn deficiency resulted in oxidative DNA damage, which means an inverse association between Zn levels and the risk of developing breast cancer (51). Consequently, the positive associations of dietary Cu, Zn, and Cu/Zn and the severity of OC are biologically plausible.

### Strengthens and limitations

Our study had strengths that are worth mentioning. The innovation is the principal strength because this is the first study to explore the association between pre-diagnostic dietary Cu, Zn, and Cu/Zn ratio intake on the severity of OC. In addition, as a cross-sectional study with a face-to-face examination of the self-administered questionnaire by well-trained researchers, the integrity and authenticity of the data are well guaranteed. Furthermore, we strictly selected and controlled the confounding factors to reduce the confounding bias.

Nevertheless, several potential limitations also deserve our attention. First, recall bias is inevitable because diet information was self-reported at baseline. However, well-trained investigators, as well as a validated FFQ, were utilized to reduce the deviation. Second, dietary Cu and Zn intake were calculated by multiplying the frequency of consumption of each food by the nutrient content of the specified portions while their dietary supplements intake of them was not included. Data from the 2010 to 2012 China Nutrition and Health Surveillance (52), a nationally representative cross-sectional study covering all 31 provinces, autonomous regions, and municipalities in China, showed that only 0.11% of the Chinese population reported using Cu supplements. About Zn, the content in dietary supplements before diagnosis had no effect on the risk and development of OC (32). Therefore, we speculated that our results might not be materially altered by the dietary supplements used. Third, we focused on the influence of diet status 1 year before diagnosis on the OC severity, but we ignored the impact of diet on disease might be a long-term process. Therefore, we adjusted the dietary change during the analysis to reduce the bias. In addition, our cross-sectional study was carried out based on the OOPS from the Shengjing Hospital of China Medical University, which is a representative hospital in the northeast region, However, the current study is a hospital-based and single-center study, selection bias may be introduced and thus generalizability of the results is largely compromised. Furthermore, residual confounding is inevitable for any observational study. Even though generally disease-related confounding factors were comprehensively adjusted in our analyses, the effect of unmeasured or unknown confounders might not be ruled out. Eventually, it is impossible to infer causality due to the cross-sectional study design.

## Conclusion

In conclusion, higher possibility of non-serous OC was associated with the higher pre-diagnosis dietary Cu and Cu/Zn ratio. The risk of poorly differentiation OC at diagnosis was significant inversely related to dietary Cu intake. Our present study provides evidence suggesting that higher pre-diagnosis dietary Cu and Cu/Zn ratio intake are conducive to reducing the severity of OC at diagnosis. However, further large prospective cohort studies are warranted to confirm these associations.

## Data availability statement

The raw data supporting the conclusions of this article will be made available by the authors, without undue reservation.

## Ethics statement

The studies involving human participants were reviewed and approved by the Institutional Review Board of the Ethics Committee of Shengjing Hospital of China Medical University, Shenyang, China (2015PS38K). The patients/participants provided their written informed consent to participate in this study.

## Author contributions

SG, Y-HZ, and LW contributed to the study design. SG, LW, SY, and XQ collected the data. J-LY and Z-YW analyzed the data. J-LY, TT, Z-YW, RW, M-HS, CG, Y-JC, SG, and LW wrote the first draft of the manuscript and edited the manuscript. All authors read and approved the final manuscript and accept responsibility for the integrity of the data analyzed.
