# Supplementary material for: Association between pre-diagnostic dietary copper, zinc, and copper-to-zinc ratio and severity of ovarian cancer
Source: Front Nutr. 2022 Nov 15;9:1003675. doi: 10.3389/fnut.2022.1003675 (PMC9705584; doi:10.3389/fnut.2022.1003675)
Supplement: Supplementary file 1 [file Data_Sheet_1.docx]

**Supplementary Table 1. Subgroup analyses for hazard ratio (HR) and 95% confidence interval (CI) for the association between dietary Copper, Zinc and Copper/Zinc ratio and histological type among 701 ovarian cancer patients**

| **Characteristics** | **Menopausal status** | | **Body mass index (kg/m^2^)** | | **Comorbidity** | |
| --- | --- | --- | --- | --- | --- | --- |
|  | No | Yes | < 24 | ≥ 24 | No | Yes |
| Serous, N (%) | 121 (61.83) | 356 (70.36) | 200 (38.61) | 117 (63.93) | 269 (68.62) | 208 (67.31) |
| Non-serous, N (%) | 74 (38.17) | 150 (29.64) | 318 (61.39) | 66 (36.07) | 123 (31.38) | 101 (32.69) |
| **Tertiles of dietary Copper intake** |  |  |  |  |  |  |
| I | 1.00 (Ref) | 1.00 (Ref) | 1.00 (Ref) | 1.00 (Ref) | 1.00 (Ref) | 1.00 (Ref) |
| II | 1.24 (0.53-2.93) | 1.11 (0.64-1.92) | 1.31 (0.77-2.23) | 1.60 (0.63-4.16) | 1.22 (0.65-2.32) | 1.13 (0.57-2.24) |
| III | 1.23 (0.37-4.04) | 2.39 (1.16-4.95) | 2.03 (0.99-4.19) | 2.45 (0.77-7.99) | 3.07 (1.34-7.17) | 1.62 (0.62-4.22) |
| **P for interaction ^*^** | 0.70 | | 0.86 | | 0.74 | |
| **Tertiles of dietary Zinc intake** |  |  |  |  |  |  |
| I | 1.00 (Ref) | 1.00 (Ref) | 1.00 (Ref) | 1.00 (Ref) | 1.00 (Ref) | 1.00 (Ref) |
| II | 1.12 (0.46-2.75) | 0.80 (0.45-1.41) | 1.03 (0.60-1.80) | 0.80 (0.30-2.10) | 0.93 (0.49-1.77) | 0.80 (0.39-1.64) |
| III | 2.62 (0.68-10.54) | 0.81 (0.34-1.94) | 0.83 (0.36-1.91) | 0.47 (0.11-1.96) | 0.72 (0.27-1.93) | 0.98 (0.33-2.93) |
| **P for interaction ^*^** | 0.42 | | 0.30 | | 0.86 | |
| **Tertiles of dietary Copper/Zinc ratio** |  |  |  |  |  |  |
| I | 1.00 (Ref) | 1.00 (Ref) | 1.00 (Ref) | 1.00 (Ref) | 1.00 (Ref) | 1.00 (Ref) |
| II | 1.07 (0.48-2.38) | 1.08 (0.64-1.83) | 1.31 (0.79-2.19) | 0.97 (0.42-2.23) | 0.99 (0.54-1.81) | 1.59 (0.82-3.09) |
| III | 1.52 (0.62-3.75) | 2.42 (1.33-4.43) | 2.32 (1.30-4.19) | 1.43 (0.56-3.65) | 2.50 (1.28-4.95) | 1.54 (0.73-3.27) |
| **P for interaction ^*^** | 0.34 | | 0.87 | | 0.91 | |

CI, confidence interval; HR, hazard ratio; Ref, reference.

***** Test for interaction was based on strata and dietary Copper, Zinc, and Copper/Zinc ratio intake.

HR and 95%CI were calculated with the use of the logistic regression model with adjusted for body mass index, education, income, menarche age, menopausal status, smoking status, drinking status, physical activity, comorbidity, parity, dietary protein, and total energy, fiber, calcium, and iron intake.

**Supplementary Table 2. Subgroup analyses for hazard ratio (HR) and 95% confidence interval (CI) for the association between dietary Copper, Zinc and Copper/Zinc ratio and histopathologic grade among 701 ovarian cancer patients**

| **Characteristics** | **Menopausal status** | | **Body mass index (kg/m^2^)** | | **Comorbidity** | |
| --- | --- | --- | --- | --- | --- | --- |
|  | No | Yes | < 24 | ≥ 24 | No | Yes |
| Well, N (%) | 21 (10.77) | 35 (6.92) | 39 (7.53) | 17 (9.29) | 31 (7.91) | 25 (8.09) |
| Moderately, N (%) | 12 (6.15) | 36 (7.11) | 33 (6.37) | 15 (8.20) | 25 (6.38) | 23 (7.44) |
| Poorly, N (%) | 162 (83.08) | 435 (85.97) | 446 (86.10) | 151 (82.51) | 336 (85.71) | 261 (84.47) |
| **Tertiles of dietary Copper intake** |  |  |  |  |  |  |
| I | 1.00 (Ref) | 1.00 (Ref) | 1.00 (Ref) | 1.00 (Ref) | 1.00 (Ref) | 1.00 (Ref) |
| II | 1.70 (0.53-5.47) | 0.75 (0.36-1.60) | 0.81 (0.39-1.69) | 0.75 (0.23-2.48) | 1.04 (0.45-2.42) | 1.09 (0.43-2.77) |
| III | 2.30 (0.48-10.96) | 0.27 (0.11-0.68) | 0.34 (0.14-0.85) | 1.17 (0.25-5.56) | 0.54 (0.18-1.60) | 0.30 (0.10-0.95) |
| **P for interaction ^*^** | 0.88 | | 0.78 | | 0.50 | |
| **Tertiles of dietary Zinc intake** |  |  |  |  |  |  |
| I | 1.00 (Ref) | 1.00 (Ref) | 1.00 (Ref) | 1.00 (Ref) | 1.00 (Ref) | 1.00 (Ref) |
| II | 0.66 (0.19-2.26) | 0.78 (0.37-1.66) | 0.42 (0.19-0.91) | 1.15 (0.32-4.06) | 0.97 (0.42-2.24) | 0.57 (0.21-1.54) |
| III | 0.34 (0.05-2.23) | 0.42 (0.13-1.31) | 0.22 (0.07-0.67) | 1.92 (0.31-12.00) | 1.76 (0.45-6.92) | 0.13 (0.03-0.51) |
| **P for interaction ^*^** | 0.85 | | 0.06 | | 0.10 | |
| **Tertiles of dietary Copper/Zinc ratio** |  |  |  |  |  |  |
| I | 1.00 (Ref) | 1.00 (Ref) | 1.00 (Ref) | 1.00 (Ref) | 1.00 (Ref) | 1.00 (Ref) |
| II | 1.07 (0.37-3.08) | 1.16 (0.56-2.38) | 1.26 (0.62-2.55) | 1.04 (0.36-3.05) | 1.36 (0.58-3.15) | 0.91 (0.39-2.10) |
| III | 1.21 (0.38-3.88) | 0.46 (0.22-0.98) | 0.59 (0.28-1.22) | 0.80 (0.24-2.72) | 0.54 (0.23-1.29) | 0.80 (0.31-2.05) |
| **P for interaction ^*^** | 0.31 | | 0.37 | | 0.55 | |

CI, confidence interval; HR, hazard ratio; Ref, reference.

***** Test for interaction was based on strata and dietary Copper, Zinc, and Copper/Zinc ratio intake.

HR and 95%CI were calculated with the use of the logistic regression model with adjusted for body mass index, education, income, menarche age, menopausal status, smoking status, drinking status, physical activity, comorbidity, parity, dietary protein, and total energy, fiber, calcium, and iron intake.

**Supplementary Table 3. Subgroup analyses for hazard ratio (HR) and 95% confidence interval (CI) for the association between dietary Copper, Zinc, and Copper/Zinc ratio and age at diagnosis among 701 ovarian cancer patients**

| **Characteristics** | **Menopausal status** | | **Body mass index (kg/m^2^)** | | **Comorbidity** | |
| --- | --- | --- | --- | --- | --- | --- |
|  | No | Yes | < 24 | ≥ 24 | No | Yes |
| ≤ 50 years, N (%) | 135 (69.23) | 123 (24.31) | 200 (38.61) | 58 (31.69) | 161 (41.07) | 97 (31.39) |
| > 50 years, N (%) | 60 (30.77) | 383 (75.69) | 318 (61.39) | 125 (68.31) | 231 (58.93) | 212 (68.61) |
| **Tertiles of dietary Copper intake** |  |  |  |  |  |  |
| I | 1.00 (Ref) | 1.00 (Ref) | 1.00 (Ref) | 1.00 (Ref) | 1.00 (Ref) | 1.00 (Ref) |
| II | 0.64 (0.24-1.68) | 1.00 (0.54-1.85) | 0.93 (0.52-1.65) | 0.86 (0.28-2.67) | 0.99 (0.51-1.94) | 0.64 (0.30-1.36) |
| III | 1.10 (0.30-4.04) | 1.35 (0.59-3.15) | 0.92 (0.42-2.03) | 3.92 (0.91-18.56) | 1.59 (0.64-4.07) | 0.82 (0.29-2.36) |
| **P for interaction ^*^** | 0.96 | | 0.64 | | 0.91 | |
| **Tertiles of dietary Zinc intake** |  |  |  |  |  |  |
| I | 1.00 (Ref) | 1.00 (Ref) | 1.00 (Ref) | 1.00 (Ref) | 1.00 (Ref) | 1.00 (Ref) |
| II | 0.75 (0.29-1.95) | 0.97 (0.511-1.85) | 0.95 (0.52-1.73) | 2.28 (0.72-7.58) | 1.28 (0.64-2.57) | 0.97 (0.45-2.23) |
| III | 0.97 (0.21-4.44) | 1.41 (0.53-3.81) | 1.10 (0.45-2.72) | 1.30 (0.26-6.57) | 2.41 (0.84-7.10) | 1.05 (0.32-3.51) |
| **P for interaction ^*^** | 0.84 | | 0.56 | | 0.57 | |
| **Tertiles of dietary Copper/Zinc ratio** |  |  |  |  |  |  |
| I | 1.00 (Ref) | 1.00 (Ref) | 1.00 (Ref) | 1.00 (Ref) | 1.00 (Ref) | 1.00 (Ref) |
| II | 0.90 (0.50-1.61) | 0.89 (0.50-1.61) | 0.98 (0.57-1.68) | 0.76 (0.29-1.99) | 1.02 (0.54-1.92) | 1.05 (0.50-2.22) |
| III | 1.11 (0.56-2.21) | 1.14 (0.57-2.29) | 0.72 (0.38-1.34) | 1.69 (0.56-5.31) | 1.15 (0.55-2.42) | 0.66 (0.29-1.48) |
| **P for interaction ^*^** | 0.31 | | 0.31 | | 0.93 | |

CI, confidence interval; HR, hazard ratio; Ref, reference.

***** Test for interaction was based on strata and dietary Copper, Zinc, and Copper/Zinc ratio intake.

HR and 95%CI were calculated with the use of the logistic regression model with adjusted for body mass index, education, income, menarche age, menopausal status, smoking status, drinking status, physical activity, comorbidity, parity, dietary protein, and total energy, fiber, calcium, and iron intake.

**Supplementary Table 4. Subgroup analyses for hazard ratio (HR) and 95% confidence interval (CI) for the association between dietary Copper, Zinc and Copper/Zinc ratio and FIGO stage among 701 ovarian cancer patients**

| **Characteristics** | **Menopausal status** | | **Body mass index (kg/m^2^)** | | **Comorbidity** | |
| --- | --- | --- | --- | --- | --- | --- |
|  | No | Yes | < 24 | ≥ 24 | No | Yes |
| I–II, N (%) | 92 (49.46) | 250 (50.81) | 211 (49.53) | 131 (51.98) | 168 (44.80) | 174 (57.43) |
| III–IV, N (%) | 94 (50.54) | 242 (49.19) | 215 (50.47) | 121 (48.02) | 207 (55.20) | 129 (42.57) |
| **Tertiles of dietary Copper intake** |  |  |  |  |  |  |
| I | 1.00 (Ref) | 1.00 (Ref) | 1.00 (Ref) | 1.00 (Ref) | 1.00 (Ref) | 1.00 (Ref) |
| II | 0.62 (0.25-1.49) | 1.10 (0.66-1.81) | 0.89 (0.53-1.47) | 0.69 (0.34-1.38) | 1.47 (0.81-2.69) | 0.76 (0.40-1.44) |
| III | 0.46 (0.13-1.59) | 1.06 (0.53-2.09) | 0.74 (0.29-1.89) | 0.92 (0.29-2.91) | 1.19 (0.54-2.64) | 0.58 (0.22-1.46) |
| **P for interaction ^*^** | 0.25 | | 0.55 | | 0.84 | |
| **Tertiles of dietary Zinc intake** |  |  |  |  |  |  |
| I | 1.00 (Ref) | 1.00 (Ref) | 1.00 (Ref) | 1.00 (Ref) | 1.00 (Ref) | 1.00 (Ref) |
| II | 1.01 (0.43-2.40) | 1.16 (0.69-1.97) | 0.93 (0.55-1.57) | 1.61 (0.62-4.24) | 1.67 (0.90-3.10) | 0.97 (0.49-1.90) |
| III | 0.92 (0.24-3.57) | 0.94 (0.43-2.08) | 1.04 (0.48-2.28) | 0.89 (0.22-3.60) | 1.61 (0.64-4.07) | 1.10 (0.40-3.01) |
| **P for interaction ^*^** | 0.38 | | 0.10 | | 0.16 | |
| **Tertiles of dietary Copper/Zinc ratio** |  |  |  |  |  |  |
| I | 1.00 (Ref) | 1.00 (Ref) | 1.00 (Ref) | 1.00 (Ref) | 1.00 (Ref) | 1.00 (Ref) |
| II | 0.62 (0.28-1.37) | 1.50 (0.93-2.42) | 1.09 (0.68-1.75) | 0.86 (0.37-1.98) | 1.36 (0.77-2.41) | 0.60 (0.32-1.11) |
| III | 0.43 (0.17-1.05) | 1.20 (0.68-2.11) | 0.68 (0.39-1.19) | 1.06 (0.42-2.69) | 0.88 (0.46-1.65) | 0.69 (0.34-1.40) |
| **P for interaction ^*^** | 0.28 | | 0.90 | | 0.64 | |

CI, confidence interval; FIGO, The International Federation of Gynecology and Obstetrics; HR, hazard ratio; Ref, reference.

***** Test for interaction was based on strata and dietary Copper, Zinc, and Copper/Zinc ratio intake.

HR and 95%CI were calculated with the use of the logistic regression model with adjusted for body mass index, education, income, menarche age, menopausal status, smoking status, drinking status, physical activity, comorbidity, parity, dietary protein, and total energy, fiber, calcium, and iron intake.
